# Parry Romberg Syndrome: When the Diagnosis of a Rare Disease Is Made in the Primary Care Setting

**DOI:** 10.7759/cureus.47397

**Published:** 2023-10-20

**Authors:** Sofia R Pereira, Rita Rodrigues, Beatriz Nunes, Bárbara D Silva, Diana Pestana

**Affiliations:** 1 Unidade de Saúde (USF) Moscavide, Agrupamento de Centros de Saúde (ACES) Loures-Odivelas, Lisboa, PRT; 2 Unidade de Saúde (USF) Amato Lusitano, Agrupamento de Centros de Saúde (ACES) Amadora, Lisboa, PRT; 3 Unidade de Saúde (USF) Conde da Lousã, Agrupamento de Centros de Saúde (ACES) Amadora, Lisboa, PRT; 4 Centro de Saúde da Madalena, Unidade de Saúde da Ilha do Pico, Ilha do Pico, PRT; 5 Unidade de Saúde (USF) Venda Nova, Agrupamento de Centros de Saúde (ACES) Amadora, Lisboa, PRT

**Keywords:** facial disfigurement, facial asimmetry, facial hemiatrophy, parry romberg syndrome, rare disease

## Abstract

Parry Romberg syndrome (PRS) is an acquired neurocutaneous syndrome with uncertain pathophysiology, and its incidence is unknown. Usually, the disease becomes apparent during the first decade of life or early during the second decade, but it can also occur in adulthood, and it is more common in females. The main feature is slowly progressive hemiatrophy (thinning or shrinkage) of the facial tissues, typically fat, skin, connective tissues, muscle, and sometimes bone. In some people, atrophy may also affect the trunk and the limbs. Additional symptoms can potentially develop in some patients, including ophthalmological, psychiatric, and neurological complications. The clinical presentation serves as a guide for the diagnosis. Treatment can demand a multidisciplinary approach (maxillofacial surgeons, plastic surgeons, ophthalmologists, neurologists, dermatologists, psychiatrists, anesthetists, and family doctors). Patients can undergo restorative plastic surgery to improve their appearance, with highly variable success rates. We present a case report of a 52-year-old man who made an appointment at the family care unit (FCU) because of a left facial hemiatrophy that started progressing two to three months before, and he was afraid it might be cancer. At the physical exam, it was possible to examine a slight hemiatrophy in two different parts of the left side of the patient's face (the nasolabial-masseter region and the temporal-malar region). The facial CT scan showed a low degree of maxillary bone resorption.

Through discussion with peers on the Family Doctor team, the diagnosis of a rare condition in the primary care setting was made possible.

This case shows the importance of being aware of a rare disease despite working as a family physician and aims to make more people familiar with this syndrome. It also raises awareness about the need for discussion of clinical cases as a team.

## Introduction

Parry Romberg syndrome (PRS), also called progressive facial hemiatrophy, is a rare, acquired neurocutaneous syndrome [1]. The main feature is a slowly progressive hemiatrophy of the facial tissues. Typical facial changes affect the maxillary region near the middle portion of the face, and they may progress to the jaw, the mouth, and the forehead. Nevertheless, patients do not present with facial weakness [2].

In some people, atrophy may also affect the trunk and the limbs, usually on the same side of the body as the facial atrophy [3]. Additional symptoms can potentially develop in some patients, including neurologic, psychiatric, and ophthalmological complications [3]. Neurologic manifestations are the most frequent systemic manifestations associated with PRS, affecting around 15% of the patients [4] and including migraine, facial pain, and epilepsy [1]. From a psychological point of view, disfigurement is often the worst symptom, leading to anxiety and depression [1]. Ophthalmological alterations may include exophthalmos and uveitis [2].

We present a case report of a 52-year-old man diagnosed with Parry Romberg syndrome, affecting only the facial tissues, identified in the primary care setting.

Our objective in presenting this case is to highlight the challenge of the diagnosis of a rare condition in the primary care setting since clinical presentation in the initial stage of the disease is usually nonspecific and the importance of case discussion with colleagues within the medical team to make the diagnosis.

## Case presentation

**Figure 1:**
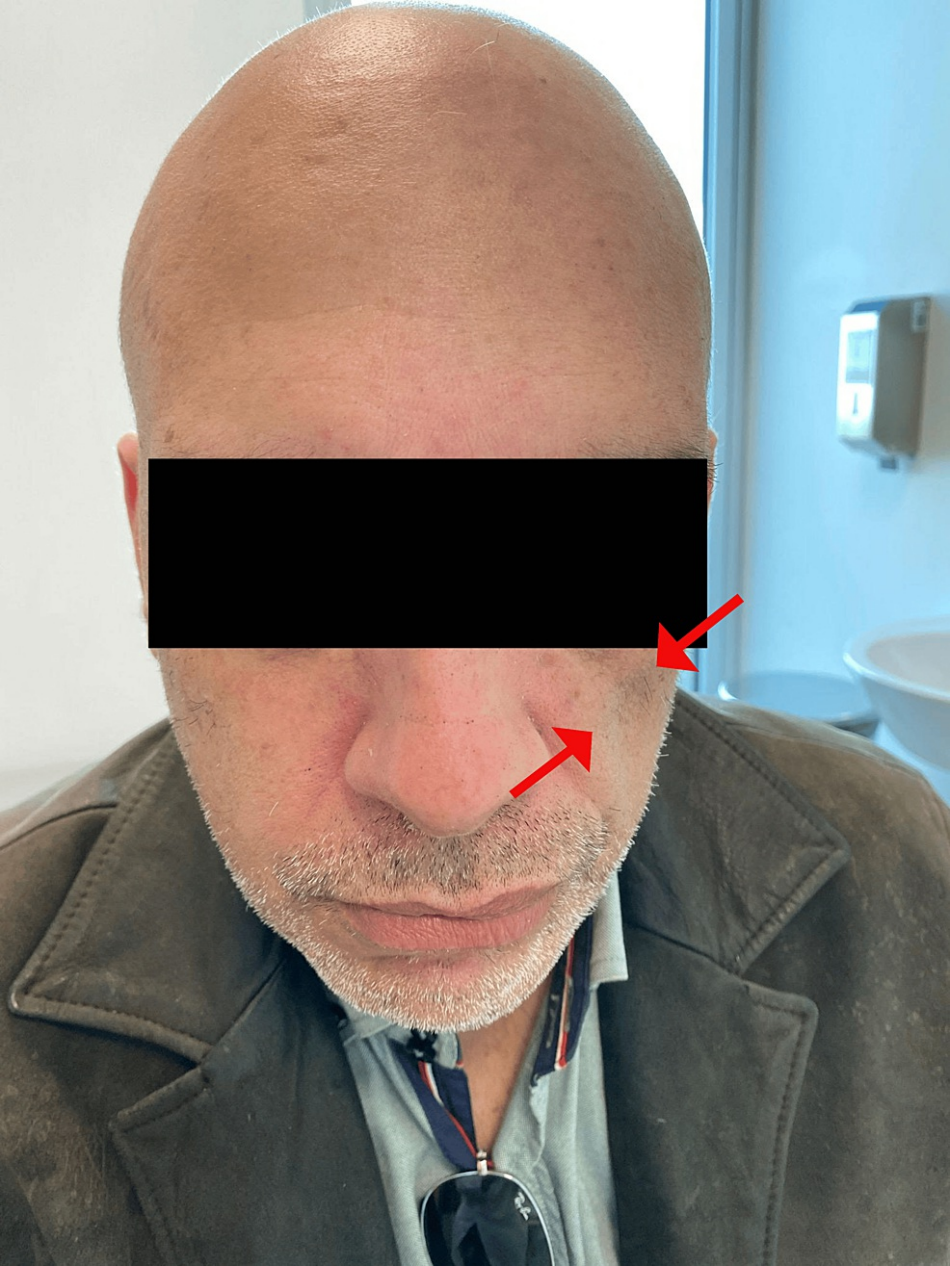
Patient's photo taken with his permission

**Figure 2:**
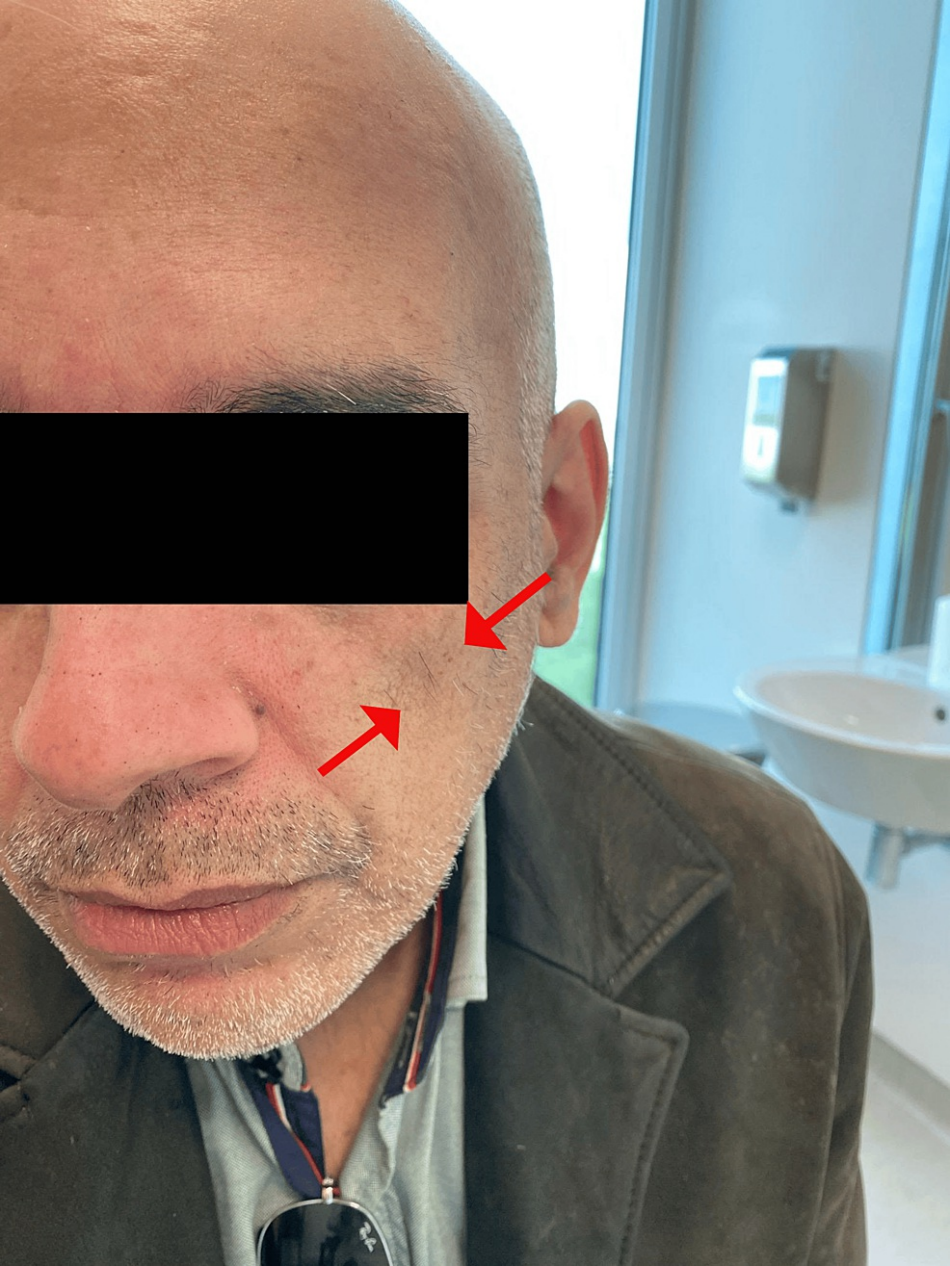
Patient's photo taken with his permission

We present the case of a 52-year-old single male who has retired from an administrative assistant job and completed secondary education. The patient attended an appointment in a family care unit (FCU) and presented with hemiatrophy of the left facial tissues, progressing over the last two to three months (Figures 1-2). He denied previous trauma, previous facial procedures or surgeries, a history of infection, febrile disease, or other associated symptoms, namely neurological. The fear of cancer motivated him to schedule an appointment.

This patient has a previous medical history of myocardial infarction in 2018, heart failure with moderately reduced ejection fraction (NYHA I), dyslipidemia, androgenetic alopecia, periodontal disease, and tooth loss due to smoking.

The patient was medicated per os with acetylsalicylic acid 100 mg q.d., furosemide 20 mg q.d., spironolactone 25 mg q.d., sacubitril 97 mg + valsartan 103 mg b.i.d., carvedilol 6,25 mg t.i.d., dapagliflozin 10 mg q.d., rosuvastatin 40 mg + ezetimibe 10 mg q.d., and pantoprazole 40 mg q.d. He is a former smoker who stopped smoking in 2018 and denied alcohol or psychotropic substance consumption. The patient denied any relevant family history.

The patient presented mild facial asymmetry with two atrophy sites on the left side of the face (in the nasolabial-masseter region and in the temporal-malar region), with no skin alterations in those same regions. The oral cavity had incomplete dentition, and there were no pathological findings in the summary neurological examination. A head CT was prescribed, and the scan revealed a mild, non-specific intensification of the cerebellar hemisphere and the superior vernix grooves. The facial CT showed no significant alterations regarding the soft tissues, such as space-occupying lesions, but evidenced a mild increase in the bone resorption of the maxillary region (images not available).

The case was discussed with the medical team of family doctors, and Parry-Romberg syndrome was highlighted as a possible diagnosis. The patient came back for another appointment, was informed of the possible diagnosis, and was referred to the hospital for a maxillofacial surgery consultation.

At the hospital, therapeutic options such as reconstructive surgery with a free fat graft and platelet-enriched fibrin application were presented to the patient. However, since general anesthesia was necessary, this treatment was contraindicated due to the coexisting cardiac problems.

The patient maintains regular follow-ups both in the hospital and the FCU, and the disease is currently stationary with no further progression.

## Discussion

Rare diseases are those that affect only a small number of individuals in relation to the general population; their diagnosis can be challenging, especially in a primary care setting [5]. There can be a significant delay between the first contact with healthcare providers and the confirmed diagnosis. In a cohort of patients with rare diseases in the United Kingdom and the United States, the diagnostic delay was an average of 5.6 and 7.6 years, respectively. This diagnosis delay is multifactorial [6].

One of these reasons is the diagnosis process itself, where clinicians consider the most prevalent and common pathologies when approaching a pattern of signs and symptoms. Enabling clinicians, especially in primary care, to identify unusual patterns and revisit diagnosis is essential to reducing the diagnostic delay in patients with rare diseases.

PRS is a rare disease, and its pathophysiology is uncertain. It is characterized by slowly progressive hemiatrophy of the facial tissues. Facial atrophy progresses slowly for several years, and frequently, it can stop progressing [1,3]. The condition was first described by Parry in 1825 and subsequently elaborated in 1846 by Romberg [4].

Usually, the disease becomes apparent during the first decade of life or early during the second, but may also develop as late as 40-50 years [7].

It is more common in females. The severity and specific symptoms are highly variable between patients, ranging from those with barely perceptible asymmetry to severe disfigurement [3]. Our patient was diagnosed at 52 years old and presented with a mild, slowly progressive disease.

In some people, atrophy may also affect the trunk and the limbs, usually on the same side of the body as the facial atrophy. More common complications described in the literature are neurologic, psychiatric, and ophthalmological [3]. Complications and limb involvement were not detected in our patient.

The physiopathology of this disease is not well understood and seems to be heterogeneous. Different theories have been proposed to explain the development of the disorder, including trauma, infections, cranial-vascular malformation, immune-mediated processes, disturbances of fat metabolism, and sympathetic dysfunction [1].

Diagnosis can be based on characteristic clinical findings, detailed patient history, and clinical evaluation without the need for further investigation [8]. Differential diagnoses include hemifacial microsomia, Bell's palsy, lipodystrophies, and a form of linear scleroderma on the scalp and forehead termed “en coup de sabre” (ECDS) [1,5]. Similar clinical and histopathological findings suggest that PRS and ECDS, although representing different clinical entities, lie on the same disease spectrum, and the overlap is described in 28-42% of cases [9]. Clinically, ECDS can present with band-like changes of sclerosis and hyperpigmentation with induration, and PRS with unilateral atrophy without induration or inflammation [8].

In our case, since the disease was acquired, hemifacial microsomia was ruled out, the patient did not present with muscle weakness, and the possibility of Bell's palsy was also excluded. Since the clinical presentation was atrophy without induration, inflammation, or hyperpigmentation, it was possible to establish the diagnosis of PRS based on clinical findings.

CT and MRI are the most common imaging methods used for better characterization of soft tissue and bone involvement and can show brain abnormalities when present [1,3]. A skin biopsy can be performed, and a lumbar puncture with an autoantibody search can be reasonable in a patient presenting with epilepsy [1,3].

Treatment demands a multidisciplinary approach (maxillofacial surgeons, plastic surgeons, ophthalmologists, neurologists, dermatologists, psychiatrists, anesthetists, and family doctors) regarding the specific presentation of the syndrome [3].

Patients can undergo restorative plastic surgery to improve their appearance, with highly variable success rates. If the disease is still active, fat injections may simply be resorbed [1]. For those with more severe and progressive disease, treatments include methotrexate, corticosteroids, cyclophosphamide, and azathioprine, but it is unclear how beneficial they are [1].

Hyaluronic acid filler use to correct skin atrophy was described in one patient with good results after two months and with no adverse effects [10]. It might be an option for our patient.

This case report aims to create awareness in the medical community about the possibility of the occurrence of this disease.

## Conclusions

In conclusion, this case report describes the diagnostic process of a case of PRS in primary care. The previous knowledge of the primary care physician of the PRS allowed for a linear diagnostic process with the correct complementary exams necessary to confirm the diagnosis and with the correct and timely referral to the secondary care setting, reducing the diagnostic delay.

In order to deal with the disfigurement caused by this disease, its management should be in secondary care settings, where there are different diagnostic and treatment methods available. Nevertheless, the family physician works as a “robust and stable bridge” between the patient and the secondary care setting, accompanying the patient through their entire life.
